# The Beige Adipocyte as a Therapy for Metabolic Diseases

**DOI:** 10.3390/ijms20205058

**Published:** 2019-10-12

**Authors:** Fernando Lizcano

**Affiliations:** Center of Biomedical Investigation, (CIBUS), Universidad de La Sabana, 250008 Chia, Colombia; fernando.lizcano@unisabana.edu.co; Tel.: +57-1-861-5555 (ext. 23907)

**Keywords:** beige adipose cell, diabetes mellitus 2, metabolic disease, obesity therapy

## Abstract

Adipose tissue is traditionally categorized into white and brown relating to their function and morphology. The classical white adipose tissue builds up energy in the form of triglycerides and is useful for preventing fatigue during periods of low caloric intake and the brown adipose tissue more energetically active, with a greater number of mitochondria and energy production in the form of heat. Since adult humans possess significant amounts of active brown fat depots and its mass inversely correlates with adiposity, brown fat might play an important role in human obesity and energy homeostasis. New evidence suggests two types of thermogenic adipocytes with distinct developmental and anatomical features: classical brown adipocytes and beige adipocytes. Beige adipocyte has recently attracted special interest because of its ability to dissipate energy and the possible ability to differentiate themselves from white adipocytes. The presence of brown and beige adipocyte in human adults has acquired attention as a possible therapeutic intervention for metabolic diseases. Importantly, adult human brown appears to be mainly composed of beige-like adipocytes, making this cell type an attractive therapeutic target for obesity and obesity-related diseases, such as atherosclerosis, arterial hypertension and diabetes mellitus type 2. Because many epigenetics changes can affect beige adipocyte differentiation from adipose progenitor cells, the knowledge of the circumstances that affect the development of beige adipocyte cells may be important to new pathways in the treatment of metabolic diseases. New molecules have emerged as possible therapeutic targets, which through the impulse to develop beige adipocytes can be useful for clinical studies. In this review will discuss some recent observations arising from the unique physiological capacity of these cells and their possible role as ways to treat obesity and diabetes mellitus type 2.

## 1. Introduction

Diabetes mellitus type 2 (DM2) is a chronic disease, the incidence of which has increased dramatically in recent years. The consequences of diabetes are devastating due to side effects that occur in the cardiovascular system [1,2]. Both DM2 and obesity has become a pandemic that has begun to appear in developing countries and in developed countries the public measures to improve lifestyle habits have not reduced significantly the incidence of these diseases [3,4]. While one approach to DM2 is based on improving lifestyle including exercise and reduction of weight. Therapeutic advances are multiple and include drugs that protecting the function of the pancreatic beta cells, increasing insulin sensitivity, rising the excretion of sugar in the urine and especially medications that reduce cardiovascular risk [5,6,7]. The current therapeutic options for obesity are limited, given the undesirable side effects presented by many of the therapies employed to date. In addition, in many countries, only a few medications have been approved and the therapeutic effectiveness of these is not as good as expected [8,9,10]. A therapy that can reduce the accumulation of calories or increase energy expenditure to improve insulin sensitivity, reduce weight and preserve the activity of the pancreatic beta cell it is desirable but has not yet been discovered. Potential new players in the area of obesity treatment are under evaluation, and like the approach to diabetes, these therapies involve drugs that enhance the balance of the satiety level in the hypothalamus and increase caloric expenditure [11,12,13,14].

Given that obesity and DM2 have precise events in common, such as energy balance, stimulation of thermogenesis and calorie expenditure, a possible therapeutic strategy could be the manipulation of the adipose tissue that controls the balance between the accumulation of energy and the production of heat. Utilizing these parameters, from this perspective, the treatment of DM2 and obesity could be through the modulation of the physiology of the adipocyte precursors [15].

In recent years, it has been observed that the adipose tissue is more dynamic than previously believed [16]. The classical white adipose tissue (WAT) builds up energy in the form of triglycerides and is useful for preventing fatigue during periods of low caloric intake. The brown adipose tissue (BAT) is more energetically active, with a greater number of mitochondria and energy production in the form of heat, which controls homeostasis during periods of low temperature and hibernation [17]. In human adults, it is believed that white adipose tissue predominates because most of BAT is seen only in the first few months of life [18]. However, it is obvious that an adipose tissue similar to brown adipose tissue can be seen in adults when they are subjected to low temperatures or sympathetic activation [19]. Despite the fact that the characteristics of this type of tissue are the same of brown adipose tissue, it is likely that these characteristics correspond to an adipose tissue variant, an energy asset that has been termed beige adipose [20,21,22].

While all adipocytes are mesenchymal in origin, an appreciable diversity arises during the process of differentiation, in fact BAT, in some way, has more in common with muscle cells of mesenchymal origin than with WAT [23,24,25,26,27]. In addition, the precursor cells of white adipose tissue can be modified with different factors to give rise to adipose tissue that is more energetically active [28,29]. Multiple factors may modulate differentiation process of beige adipocytes from adipocyte precursor cells. Extracellular signals include the activation of the sympathetic nervous system, the cells of the immune system and epigenetic variations [30] that influence the transcription of specific genes [31].

## 2. The Origin of Adipocytes

Adipocytes are derived from mesenchymal stem cells (MSCs), which can be neuroectodermal or mesodermal depending on where the fat body originates; differentiation of adipocytes requires a committed pre-adipocyte progenitor [32]. Visceral white adipose tissue is primarily derived from the lateral plate mesoderm, brown fat is largely produced by the paraxial mesoderm, and cranial white adipocytes from the neural crest [33,34]. Beige fat arises from white fat (precursors or mature cells). Despite this common origin, beige fat is thermogenic, like brown fat, so it plays a different metabolic role than white fat and has a correspondingly different transcriptional program than white adipocytes [35]. The beige adipocyte is a type of adipose cell described by the ability to induce these cells to produce heat and increase energy expenditure. The beige adipocyte can be considered phenotypically as a fat cell that possesses characteristics between those of the white fat cell, an accumulator of energy, and the brown cell, which produces heat [36] (Table 1).

The beige adipocyte biogenesis, also called beige adipogenesis or (beigeing), is induced with chronic exposure to the external cues such as cold, adrenergic stimulation, long term treatment with PPARγ agonists, among others. Beigeing is a temporary adaptive response which lasts even after the dissipation of external environmental signals [37,38]. The origin of the beige adipocyte is complex; some beige adipocytes arise in epididymal white fat from precursors that express platelet-derived growth factor receptor alpha PDGFRα, CD34, and spinocerebellar ataxia type 1 (SCA1) proteins [39,40,41,42,43,44]. Beige adipocytes may be obtained from the myogenic factor 5 (*Myf5*)-negative precursors of inguinal white fat tissue. However, a group of these adipocytes originated from *Myf5*-positive precursors have been reported [43]. Recently, it has been described that some beige adipocytes are myosin heavy chain 11 (*Myh11)*-positive, which is a selective marker of smooth muscle cells [45]. All of these results indicate that beige adipocytes have a cellular origin different from the classical brown adipocyte.

Another feature of beige adipocytes is the capacity to have a flexible phenotype. While development of beige adipocytes is highly inducible from precursor cells, there is evidence that mature white fat cells can be changed to beige adipocytes through specific factors [46,47]. Whether this phenomenon represents a real transdifferentiation, direct transformation of white adipocyte cells to a beige adipocyte cells, or corresponds to a beige adipocyte that was probably previously hidden among the white fat cells is a matter of debate [48]. One of the issues involving any special assessment in human adults is whether the adipocytes in which thermogenic properties have been detected are in reality brown adipocytes or beige adipocytes [49,50]. According to recent studies, most of these adipocytes in the adult are considered to possess the characteristics of beige adipocytes. However, in zones like the posterior part of the neck still there are adipocytes that conserve brown phenotype [24,49,51,52,53].

Adipocyte turnover and ultrastructural studies suggest that adipose progenitor cells (APCs) continually supply the tissue with new adipocytes throughout the life span of the organism. In humans, the number of fat cells stays constant in adulthood in lean and obese individuals, even after marked weight loss, indicating that the number of adipocytes is set during childhood and adolescence [54]. A common notion in accordance with recent researches is that adult APCs reside in the stromal vascular fraction (SVF). Notably, a similar population of murine SVF cells has been identified that have adipogenic potential, but these APCs express PPARγ [55,56]. Using genetic-tracing methodologies, it was found that PPARγ-expressing APCs are critical for adipocyte formation in vitro and in vivo [55,57]. In vivo tracking of PPARγ+ cells indicated that these cells reside within the blood vessel walls of white adipocytes. In line with a vascular residency, these APCs resemble mural cells (aka: pericytes and vascular smooth muscle cells) because they express several mural cell markers, such as PDGFRβ and alpha smooth muscle actin (SMA). Smooth muscle genetic fate-mapping studies have suggested cells marked by Myh11, PDGFRβ, and SMA can generate beige adipocytes in response to cold exposure. SMA+ perivascular cells generated 50–70% of new beige adipocytes after 1 week of cold exposure. Remarkably, blocking adipogenesis within SMA+ cells or ablating SMA+ cells led to the failure of cold-induced beige adipocytes and mice were unable to either defend their temperature or lower plasma glucose [58].

## 3. Adipose-Derived Stem Cells (ADMSC)

The ADMSC has a great challenge for the treatment of systemic diseases that are of an inflammatory origin. Although the mechanism by which these cells can improve the conditions of patients are not fully elucidated. Evidence is accumulating that many of the immunomodulatory effects of ADMSCs are mediated by host cells. It has been demonstrated that the induction of apoptosis of intravenously infused ADMSCs and the subsequent engulfment of ADMSCs by phagocytic cells is crucial for the therapeutic effect of MSCs in graft versus host disease [59,60].

This hypothesis suggests that maximal therapeutic effects of ADMSCs can be obtained not by optimizing the migratory capacity and secretome profile of ADMSCs, but by generating ADMSCs that are optimally capable of inducing an immune regulatory and regenerative phenotype and function in phagocytic cells [61]. ADMSCs are under consideration as a treatment for a wide variety of conditions and the types of condition determines the route of administration of the cells. For most immunological disorders, intravenous administration has been the route of choice whereas for bone repair purposes. ADMSCs are seeded on transplantable scaffolds or administered as in vitro generated cartilaginous templates that undergo osteogenic differentiation after implantation [62]. Intralesional administration of 120 million allogeneic expanded ADMSCs (darvadstrocel, formerlyCx601) over control in the treatment of complex perianal fistulas in Crohn’s disease patients has showed a significant beneficial effect [63].

## 4. Beige Thermogenesis

In mammalian cells, the free energy required for life is provided by reduced substrates. Mitochondrial oxidative phosphorylation dominates metabolism in mammalian cells, transducing this free energy into displacement of the ATP to ADP+Pi. Remarkably, only a fraction of the thermal energy flow of substrate oxidation is conserved in the free energy of the displaced equilibrium; most is lost as heat. Therefore, in cells where the mitochondrial respiratory chain dominates oxidative metabolism, the rate of mitochondrial respiration is the major determinant of heat production. Since brown and beige adipose tissue metabolism is predominantly oxidative, thermogenesis in these cells is effectively controlled by manipulation of rate limiting steps in respiration [64].

Substrate oxidation by the mitochondrial respiratory chain drives an electrochemical proton gradient across the mitochondrial inner membrane. The protonmotive force (Dp) generated by mitochondrial respiration drives protons back into the mitochondrial matrix through the ATP synthase, providing energy for the reaction ADP+Pi/ATP [65].

UCP1 is required for proton leak in brown and beige adipocytes. The mitochondria of the brown and beige adipocytes show a lack of respiratory control (relationship between ATP/ADP) which can be redirected by the balance between the purine nucleotides and the free fatty acid sequestration [66]. Experiments carried out in mitochondria of brown fat cells have shown that an increase in the concentration of free fatty acids leads to a higher conductance of protons by UCP1. On the other hand, purine nucleotides are capable of inhibiting the action of UCP1. In this process, purine nucleotides bind to the cytosolic face of UCP1 and appear to hide the pathway of proton translocation. Under basal conditions the inhibition made by purine nucleotides predominates and proton leak mediated by UCP1 is reduced [67]. Basal proton leak accounts for 20–30% of the resting metabolic rate of hepatocytes and up to 50% of the respiration of skeletal muscle of a rat. Considering the high metabolic activity of the liver and the large proportion of skeletal muscle relative to body mass, basal proton leak contributes significantly to basal metabolic rate of a resting mammal at thermoneutrality in the postabsorptive state [68].

However, external stimulation reveals the thermogenic capacity of beige and brown cells, both in culture and in vivo. In experimental mammals and humans, environmental cold is a powerful trigger of brown/beige fat respiration. This stimulus engages cold sensitive thermoreceptors, which transmit afferent signals to the hypothalamus and brain stem, leading to the release of noradrenaline from postganglionic sympathetic nerves that innervate brown adipocytes leading to the release of noradrenaline from postganglionic sympathetic nerves that innervate brown adipocytes [24]. Noradrenaline acts on adrenoreceptors on the adipocyte plasma membrane, which ultimately results in the release of free fatty acids from stored triglycerides. Upon adrenergic stimulus that results in activation of the brown (and white) adipocyte lipolytic cascade, leak respiration increases in a UCP1-dependent manner [69].

Thermogenesis has proven to be independent of free acidosis lipolysis. Recently, a thermogenic effect of reactive oxygen species (ROS) has been observed. Genetic or pharmacological elevation of adipocyte ROS levels, or resulting oxidation of cellular thiol status, is sufficient to drive elevated adipocyte thermogenesis. Moreover, activation of thermogenesis in mouse BAT by applying either thermal stress (4 °C) or β-adrenergic stimulus results in elevated levels of mitochondrial superoxide, mitochondrial hydrogen peroxide, and lipid hydroperoxides. A major thermogenic action of mitochondrial ROS is likely mediated through protein cysteine modification [70]. A recent study identified a mechanism whereby substantial and selective accumulation of the mitochondrial metabolite succinate can act as a potent molecular source for thermogenic ROS in brown and beige fat [71].

Aside the strong evidence of the role of UCP1 in thermogenesis, substantial work has focused on whether presence of the UCP1 protein alone is necessary to drive adipocyte thermogenesis [72,73]. In fact, studies in UCP1-KO mice have shown that the production of thermogenesis could have diverse routes. It was demonstrated that the cold sensitivity of inbred UCP1-KO mice can be completely rescued by crossing this strain to mice expressing transgenic PRDM16 driven by a *Fabp4*/*aP2* promoter [72]. Remarkably, UCP1-KO mice are resistant to diet-induced obesity at sub-thermoneutral temperatures, presumably via activation of poorly defined alternate routes of energy loss in the absence of UCP1 [74]. However, there is controversy about the possibility of thermogenesis being UCP-1 independent. In this sense it is likely that mice with UCP-1KO have a thermogenesis induced by muscular activity that produces shivering thermogenesis. Creatine has been related with metabolism and mitochondria heat production. Recent observations suggest the presence of a mitochondrial substrate cycle that is regulated by creatine to drive thermogenic respiration [75,76]. The thermogenic action of creatine seems to occur only when ADP is limiting, which is the expected parameter of the physiological cellular state. Although the mechanism by which creatine influences mitochondrial metabolism is yet to be established. Different animal models with genetic modifications have shown that reducing creatine may predispose to obesity [75,77,78]. A recent analysis of 18F-FDG PET/CT scans in human subjects demonstrated that renal creatinine clearance was a significant predictor of total activated human BAT [79]. Since creatinine is a direct product of phosphocreatine metabolism, these results are consistent with activation of creatine-dependent thermogenesis in human BAT and suggest that creatinine may be used as a biomarker of human BAT activity.

## 5. Epigenetics Modifications in Adipose Cells

### 5.1. Epigenetic Regulation by DNA Methylation

DNA methylation plays a critical role in thermogenic adipose development and gene regulation. There are many regions involved in thermogenic adipogenesis that are controlled epigenetically, as global inhibition of demethylation greatly impacts general adipogenesis. The increase of DNA methylation plays a complex role in adipogenesis. While the inhibition of DNMTs (DNA Methyltransferases) in early stages enhanced adipogenesis [80,81]. During later stages DNMTs inhibition does not affect the adipogenesis processes, this is observed in multiple tissue culture models including multipotent C3H10T1/2, ST2 cells, and pre-white adipocytes 3T3-L1. The expression of ten-eleven translocations proteins (TETs), the modulator of DNA demethylation, is upregulated in tissue culture models of both white and brown adipogenesis [82].

PR domain containing 16 (PRDM16) is a key development transcriptional regulator that commits progenitors to the brown adipose heredity and maintains brown adipocyte identity. PRDM16 is enriched with CpG sites around its transcription start site, and hypomethylation at three specific regions of its promoter, likely mediated by the TET proteins, leads to increased *Prdm16* expression during brown adipogenesis [26]. Previous studies found that white adipogenesis has more hypermethylation overall than brown adipogenesis, and it is located mostly at intronic and intergenic regions. On the other hand, brown adipocytes have hypomethylated exonic regions that are significantly enriched for genes involved in brown fat functions such as the mitochondrial respiratory chain and fatty acid oxidation [83].

Notably, several Hox transcription factors are differentially methylated, some of which are linked to adipogenesis and diabetes. For example, Hoxc9 is a well-established adipocyte marker, and Hoxc9 and Hoxc10 promoter methylation is inversely correlated with their gene expression in BAT [84,85,86]. Brown adipocyte-specific *Ucp1* expression is associated with reduced CpG methylation at the *Ucp1* enhancer and can be further reduced by DNMT inhibitor in brown adipocyte HIB1 cells [87]. Peroxisome proliferator-activated receptor gamma coactivator-1 alpha (PGC-1α) is required for the cold-inducible expression of *Ucp1*. PGC-1α is a transcriptional co-activator that regulates genes involved in energy metabolism, and its methylation increase in the context of insulin resistance and exercise in tissue like skeletal muscle [88].

Transgenerational effect occurs when the epigenetic modification maintaining to subsequent generations without the presence of trigger factors. Accumulating evidence supports that DNA methylation plays an important role in the heritability of obesity and other metabolic disorders. Cold exposure in males, but no female, prior to conception results in increased cold tolerance and improved whole-body metabolism in male offspring in association with enhance expression of *Ucp1* in brown adipocytes [89]. Other studies showed that neonates born to obese wild-type mice have reduced brown adipose activity. Obese mother induces hypermethylation of *Pparγ* promoter region in the offspring with persistently lower *Pparγ* expression [90]. These mice have reduced *Prdm16* expression, in association with a reduced α-KG (alfa-ketoglutarate) level, due to DNA hypermethylation at *Prdm16*. As a result, *Ucp1* expression is reduced in these offspring, impairing the ability to maintain body temperature in response to cold [91].

### 5.2. Epigenetics and Mechanisms of Chromatin Modification

Histones are subject to various post-translational modifications such as acetylation, methylation, phosphorylation, and ubiquitination and thus contribute to the regulation of chromatin states and transcriptional activities. The chemically stable characteristics of methylated histone would contribute to the cellular memory of external stimuli by maintaining the transcription levels of adaptive genes even after the dissipation of environmental cues. It is speculated that histone methylation would be involved in the regulation of beige adipogenesis, which is one of the mechanisms for adaptation to the external environment (e.g., coldness). During the differentiation process of beige adipocytes, there are several factors that modify the chromatin and thus determine the function of genes that increase caloric expenditure and determine a beige adipocyte phenotype. recent studies have revealed that the cell fate of beige adipocyte is regulated by diverse histonemethyl-modifying enzymes such as JMJD3 (also known as KDM6B) [22], euchromatic histone-lysine N-methyltransferase 1 (EHMT1) (also known as GLP) [23], Jumonji domain containing 1A (JMJD1A) (also known as JHDM2A or KDM3A) [24], and LSD1 (also known as KDM1A) [92,93,94,95,96].

The histone lysine demethylases KDM4A/JMJD2A, -JMJD2C, and KDM3 A/JMJD1A may interfere with the differentiation of adipocytes by different mechanisms. KDM4A and KDM4Ccan modify the expression of genes controlled by the nuclear receptor PPARγ [97,98,99]. KDM3a catalyzes the removal of methyl-residues from H3K9me1/me2. While KDM3A regulates beige adipocytes biogenesis through its demethylase activity, it regulates BAT function by the mechanism independent of its enzyme activity [94]. KDM3A bides within the SWI/SNF complex in the chromatin and controls the activity of the α1- adrenergic receptor [100]. Mice with a *kdm2a* knockout have an obese phenotype due to the increased oxidation of fatty acids [101,102].

The histone methyltransferase EHMT1 forms a complex with PRDM16 and is required for the determination of the production of the beige lineage [93]. EHMT1 increases the production of UCP1, and the deletion of this EHMT1 causes insulin resistance and obesity in mice, while a haploinsuffiency is related to obesity and insulin resistance in humans [103,104].

Histone deacetylase 1 (HDAC1) negatively regulates the thermogenic program of brown adipocytes [105]. Coordination between the inhibition of HDAC1 and the activation of the histone demethylase Lysine-specific demethylases 6 (KDM6B/JMJD3A) and KDM6A/UTX activates brown adipocyte genes and prevents the appearance of obesity [106,107].

**Figure 1:**
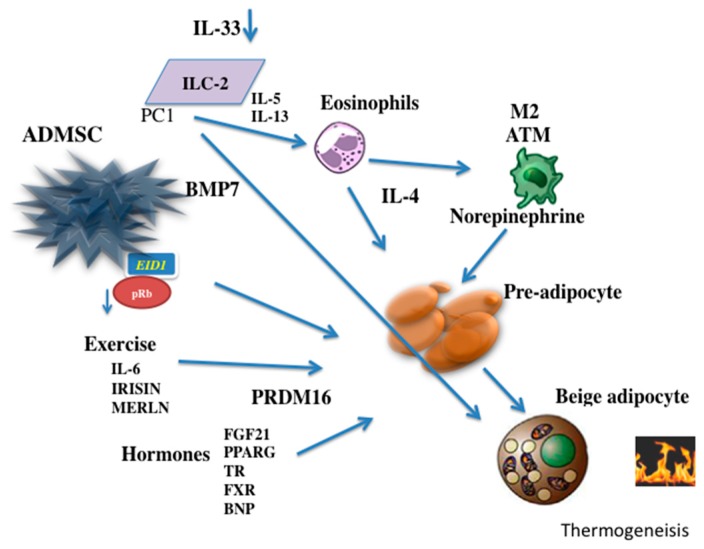
Determining factors in differentiation of adipose cell Beige. The adipose mesenchymal cells can be influenced by the retinoblastoma protein (pRb) and take decision to differentiate into fat cells when pRb is blocked. The EID1 protein among others can determine the differentiation of beige cells adipocytes from mesenchymal stem cells. BMP7 triggers production of mesenchymal adipose cells to brown adipose cells. Both exercise and some hormones can increase the capacity of adipose stem cells differentiate into beige adipocytes. Recently, it has been observed that cells of the innate immune system type 2, can secrete interleukins stimulating the production of IL-4 by eosinophils and norepinephrine production through the type 2 macrophage. IL-33 has the ability activating the differentiation of adipocytes directly Beige. ADMSC: Adipose Mesenchymal Stem cell; PC1: Prohormone Convertase 1;ILC-2 Group 2 innate lymphoid cells; BMP7: Bone morphogenic protein 7; EID1: EP300-interacting inhibitor of differentiation 1; M2 ATM: Adipose tissue type 2 macrophage; FGF21: Fibroblastic growth factor 21; PPARγ: Peroxisome proliferator-activated receptor gamma; TR: Thyroid receptors; FXR: Farnesoid X receptor; BNP: Brain natriuretic factor.

The retinoblastoma protein (pRb) is a regulator of the differentiation of mesenchymal cells to different levels [108]. A deficiency in pRb increases the differentiation of the mesenchymal cell precursors into the brown adipocyte lineage with a reduction in the differentiation into osteoblasts or white adipocytes [109]. In addition, a blockage of pRb activity can control the decision of adipocyte precursors to progress toward the development of beige adipocytes. EP300 Interacting Inhibitor of Differentiation 1 (EID1/CR1) can reduce the activity of pRb and induce differentiation to beige adipocytes [110,111]. p107, a cell-cycle regulator that belongs to the pRb family, has demonstrated an important role in the decision of mesenchymal stem cells in the differentiation of beige adipocytes [112] Figure 1.

### 5.3. Epigenetics Changes by Non-Coding RNA

The regulation in gene expression is also established by non-protein-coding transcripts of long and small RNAs (ncRNAs), which represent almost 90% of our RNA.

In recent years, an explosion in the identification of ncRNA and their functions were observed, yet one only began to understand the complexity of this new regulatory RNA world, in particular how ncRNAs govern various aspects of gene expression and their involvement in diseases. MicroRNAs (miRNAs) are a class of short non-coding RNAs that alter the expression of genes. Despite the fact that the main effect of miRNAs is the inhibition of the translation machinery, an increase in activity has been observed in some cases. A large number of miRNAs have demonstrated the ability to regulate the differentiation of beige adipocytes from precursor cells. However, only one group has shown a specific effect with possible clinical relevance. Some miRNAs have the ability to negatively regulate the activity of PRDM16, including miR-133, miR-193b, and miR-365 [113,114,115]. miR-93 acts as a negative regulator of adipogenesis by influencing adipocyte precursors via the modulation of sirtuin 7 (Sirt7) [116].

Other miRNAs, such as miR-196a, can increase the production of beige adipocytes by blocking the expression of Homeobox C8 (HoxC8), which negatively regulates the activity of CCAAT/enhancer-binding protein beta (C/EBP-β) [117]. The influence of miRNAs seen in the repression of the activity of molecules such as phosphodiesterase 1β (PDE1β) and receptor interacting protein 140 (RIP140) by miR-378 and miR-30, which induces the development of beige and brown adipocytes [118,119]. miR-34 may suppress the differentiation of beige adipocytes by reducing the activities of sirtuin 1 (Sirt1) and fibroblast growth factor 21 (FGF21) in mice [120]. So far, miR-26a and miR-26b are the first human miRNAs characterized in depth in beige/brown adipogenesis and were found to be able to shift adipocyte differentiation from white to beige via induction of UCP1 expression, increase in mitochondrial density, morphological changes in mitochondria towards brown adipocyte characteristics, and via an increase in energy expenditure miR-26 mediates these functions at least partially via its direct target ADAM metallopeptidase domain 17 (ADAM17), as its knockdown in mice causes a lean, hypermetabolic phenotype [121,122].

Long noncoding RNAs (lncRNAs) are a unique class of transcripts that share similarities with mRNA with regard to their transcriptional regulation and biogenesis, but lack protein-coding potential, long noncoding miRNAs, such as BATE1 and Blnc1, are required for the formation of beige and brown adipocytes through the production of related nucleoproteins that influence the activation of thermogenic genes [123].

Compared to control, adipocytes overexpressing hBLNC1 exhibited significantly increased mRNA expression of *Ucp1*, *Elovl3*, and *Cox7α1*, genes associated with brown and beige adipocyte thermogenesis. Interestingly, the mRNA levels of several transcriptional regulators of thermogenic gene program, including *Pgc1α*, *Pparα*, and *Prdm16*, were also significantly increased by hBLNC1 [124,125]. In vivo models showed dual role of Blnc1 in driving cold-induced thermogenesis and restricting obesity-associated brown fat whitening [126].

## 6. Induction of Beige Adipocyte by Cold

The primary inducer of beige adipocytes is a reduction in temperature, this effect is obtained mainly through the activation of sympathetic nervous system. The general consensus is that cold stimulates a greater release of catecholamines by the nervous system, an event that stimulates thermogenesis through the activation of the protein kinase A (PKA) and p38 mitogen-activated protein kinases (p-38 MAPK) pathways followed by the activation of UCP1 and phosphorylation of the specific factors PGC-1α, cAMP response element-binding protein (CREB) and activating transcription factor 2 (ATF2) [18,19,127,128].

**Figure 2:**
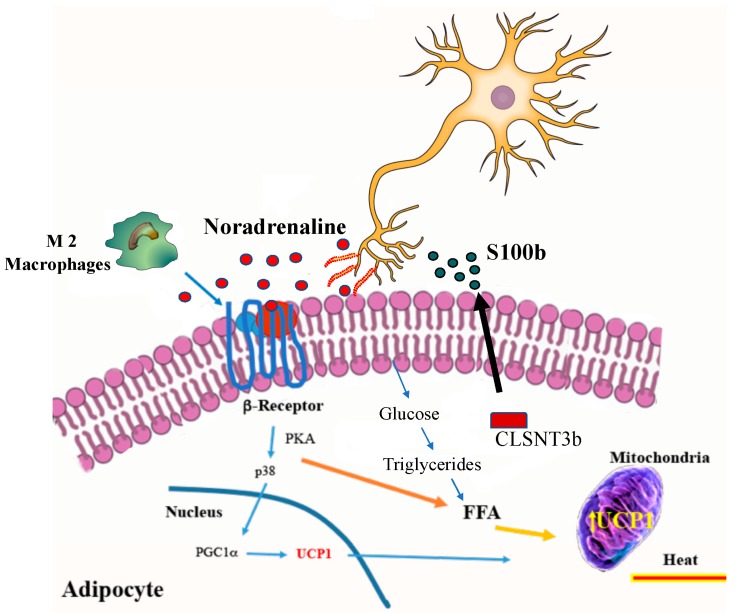
The effects of cold on the induction of thermogenic active adipocyte. The secretion of noradrenaline directly from nervous sympathetic system, or indirectly through catecholamine secretion by macrophage type 2 can stimulate glucose uptake in fat cells and improve the regulation of carbohydrate. Both glucose and triglycerides are used by UCP1 protein to increase thermogenesis. Calsyntenin 3β (CLSTN3β) that is expressed in beige and brown adipocytes aids the secretion of a growth-factor protein, S100b that facilitates the growth of projections from neurons and noradrenaline secretion. FFA: Free fatty acid; M2: Macrophages 2, SNS: Sympatic Nervous System; PKA: Protein Kinase A; UCP1: Uncoupling protein 1; PGC-1α: Peroxisome proliferator-activated receptor-gamma coactivator alpha 1; CLSTN3β: Calsyntenin 3β; S100b, S100 calcium-binding protein b.

Additionally, increase of norepinephrine after cold stimulation is associated with the immune system. In fact, a greater number of type 2 macrophages with capacity to produce catecholamines have been observed in the subcutaneous fatty tissue after exposure to the cold [22,129] Figure 2.

The cold as an inducer of the beige adipocyte has two functions, an initial effect that the cold has on the precursors of the adipocytes and the role that the cold plays in the mature cell during the process of beigeing. While the cold effect in part may be mediated by IL-4 and its receptor IL-4Ra in the adipocyte precursors; in mature adipose cells is observed a reduction in the IL4Ra and probably this effect may include diverse mediators, including IL-33 and Met-enkephalin [130,131].

Rich environmental stimuli (physical and social stimulation) play important roles in the dynamics of beige adipocyte development. An environmental stimulus may produce an increase in the secretion of catecholamines via the hypothalamic secretion of Brain-derived neurotrophic factor (BDNF) [132]. The level of the neuron growth factor inducible VGF is increased in rich environments, and VGF appears to act as a mediating factor in the BDNF pathway [133]. Recently was observed that CLSTN3β, a protein expressed in brown and beige adipocytes may mediate, in part, the sympathetic effect on adipocytes. CLSTN3β and S100b are abundantly and selectively expressed in thermogenic adipocytes, which provides strong support for their roles as adipocyte-derived neurotrophic factors. It is probably that NGF or BDNF also contribute to adipose innervation [134]. CLSTN3β expression is regulated by lysine demethylase 1 LSD1/KDM1A protein which modulates the beigeing process [135]. An increase in CLSTN3β in the endoplasmic reticulum increases S100b secretion. Secreted S100B appears to exert neurotrophic effects on sympathetic fibers in brown adipose tissue contributing to the larger sympathetic innervation of this latter relative to white adipose tissue [136]. Under these conditions CLSTN3β could become in therapeutic target that mediates the effect of cold on fat cells.

## 7. Influence of Physical Exercise

Physical exercise has an effect on beigeing based on observations in animals. Apparently more than the intensity of the exercise the effect is more related to the duration of physical activity [137,138]. There are multiple hypotheses that have been brought forth to explain the mechanisms responsible for the exercise-induced increase in beigeing [139]. Studies have shown that beigeing occurs in response to increased secretion of the hypothalamic brain-derived neurotrophic factor (BDNF) during exercise [132]. The main mediator of this process seems to be PGC-1α, which influences myogenesis, mitochondria and oxidative phosphorylation [140,141,142]. Some proteins produced in the muscle can influence the metabolism of the fat cell. The derived from fibronectin type III domain-containing protein 5 (FNDC5), Irisin and Meteorin-like protein precursor (METRNL) can lead to the process of beigeing in different ways [143,144]. While Irisin regulates the expression of specific genes in beige adipocytes [145], METRNL increases the activation of type 2 macrophages through eosinophils [146]. The pro-inflammatory interleukin IL-6 can also increase the process of beigeing and induce an increase in calorie expenditure [147].

Three weeks of wheel cage running in mice significantly decreased the abundance of triacylglycerols (TAGs), phosphatidylcholines (PC) and cholesterol esters (CE) and increased specific molecular species of PC and phosphatidylethanolamines (PE) in brown adipocytes. Exercise decreased expression of genes involved in phospholipid metabolism (*Agpat3*, *Gpd1*, *Lgpat1*, *Ptdss2* and *Pld1*) and fatty acid biosynthesis (*Acaca*, *Scd1*, *Agpat3*, *Dgkd* and *Mlxipl*). The decrease in genes involved in fatty acid biosynthesis corresponded to a decrease in overall TAGs in brown adipocytes. These data indicate that exercise causes significant adaptations to the brown adipocytes lipidome, although the physiological effects of these changes on insulin sensitivity and glucose tolerance are still topics of investigation [148].

Lipid control in adipocytes can be changed during exercise. The size of the adipocytes are diminished and there is a reduction in the content of 18:1 (oleic acid) monounsaturated fatty acids and increase in linoleic acid (18:2 n-6) content in subcutaneous adipose tissue after chronic training in humans [149]. The decrease in the level of 18:1, the main monounsaturated FA (MUFA), might be associated with reduced activity of stearoyl-CoA desaturase (SCD1) in adipocytes [150]. Since metabolic disorders were shown to be associated with enhanced synthesis of 18:1 and other MUFA by SCD1 [151] a post-exercise decrease in adipose tissue content of 18:1 may be considered a favorable change. After 6 months of increased physical activity contributed to a significant increase in 18:2 n-6 in overweight elderly subjects, while no such effect was observed in untrained controls [152]. Previously was shown that training decrease in palmitoleic acid (16:1) and an increase in stearic acid (18:0) content, comparing to untrained controls [153]. Although the evidence is subtly short from human suggests that chronic exercise may contribute to a decrease in 18:1 content, with concomitant increase in 18:2 n-6 and 18:0. On the other hand it is very feasible that the it is likely that a product of anaerobic exercise such as lactate can influence the process of beigeing; this possibility requires future study [154].

## 8. Role of Interleukins

Obesity has been considered to be a condition of slight chronic inflammation, and interleukins can influence the function of fat cells in diverse ways. Pro-inflammatory interleukins that are increased in obese people, such as TNF-α, IL-1α and IL-6 secreted by type I macrophages, induce undesirable effects leading to cardiovascular complications in patients with obesity [155,156,157,158]. In contrast, in thin subjects and with specific stimuli, it is possible to generate a change in the population of macrophages by increasing the number of anti-inflammatory type II macrophages [159]. These macrophages may maintain sensitivity to insulin and remodeling of the extracellular matrix [160]. Type II macrophages are activated by T-lymphocytes and eosinophils [161,162]. In mice, Th2 lymphocytes secrete the anti-inflammatory interleukin IL-10, which improves insulin sensitivity by blocking the action of TNF-α. In addition, the eosinophils that migrate to the adipose tissue can maintain the activity of the M2 macrophages via the secretion of IL-4 and IL-13 [163,164,165]. The presence of type 2 innate lymphoid cells (ILC2s), which act as T helper lymphocytes and produce IL-4, IL-5 and IL-13, has attracted particular interest because a reduction of these cells in adipose tissues is associated with obesity in mice and humans [166]. Recently it has been observed that IL-33 is required for the maintenance of ILC2s in the white fat cells and for the development of the beige adipose phenotype [167]. IL-33 can induce the production of IL-4 by eosinophils and increase the production of Met-enkephalin by ILC2s [130,168].

## 9. Endocrine Factors and Metabolites in Beige Adipocytes

There are a significant number of endocrine factors that have the ability to regulate the occurrence of beige and brown adipocytes. Factors such as BMP7 [169,170], BMP8b [171], FGF21 [172,173], prostaglandins [174,175], natriuretic peptides [176] and β-aminoisobutyric acid (BAIBA) [142] can influence the differentiation of beige adipocytes. All of these factors, which are capable of increasing caloric expenditure by various mechanisms, have protective effects regarding obesity in animals fed a high-caloric diet and improve glucose homeostasis and insulin sensitivity [177].

The activation of the nuclear receptor PPARγ has a strong effect on the differentiation of adipose cells and is involved in the differentiation of all types of fat cells. PPARγ agonists have been used clinically to improve insulin sensitivity; however, undesirable side effects have limited the therapeutic use of these compounds [178,179]. Recently, selective agonists of PPARγ have been shown to control the expression of genes through enzymatic modifications such as phosphorylation or deacetylation [180,181,182]. The mechanism by which some ligands of PPARγ induce the transcription of genes involved in the process of differentiation of the beige adipocytes includes the activation of Sirt1, a histone deacetylase NAD^+^-dependent protein, which might be influenced by PPARγ itself and combine with PRDM16 [183,184]. It has been observed that an increase of Sirt1 in adipose tissue improves obesity by increasing thermogenesis.

Thyroid hormones have a known influence on thermogenesis in view of their effect on the metabolic functions of all body cells. Most of the actions of thyroid hormones are mediated by the union with intracellular receptors belonging to the nuclear receptor superfamily [185,186]. The major effect of thyroid hormones is exerted by T3 (triiodothyronine), which is converted from T4 by the deiodinase proteins [187]. There are three known deiodinases with different distribution in the body. The deiodinases with relevant physiological effect are the type 1 deiodinase (DIO1) found in almost all body tissues and the type 2 deiodinase (DIO2) found at the level of hypothalamus, pituitary gland and brown adipose tissue [188].

T3 positively regulates mitochondria function and induces mitochondrial biogenesis, this action is in part mediate for the increase of (PGC-1α). The PGC-1α gene contains a thyroid hormone responsive element T3 rapidly induce PGC-1α expression [189,190].

Facultative thermogenesis is T3 dependent, as illustrated by the fact that hypothyroid rats do not survive after few hours of cold exposure [191]. During cold exposure, an intense activity of sympathetic flux and norepinephrine (NE)/cAMP/protein kinase A signaling activates DIO2 activity in BAT [192] to increase the local T3 production. Local T3 controls the expression of many proteins implicated in the turnover of lipid metabolism [193], and lipogenesis is very important for the maintenance of lipid stores that will be used to provide free fatty acids to activate UCP1. Like hypothyroid mice, the DIO2 deficient mice are unable to survive in low temperature for a long period of time. Apart from the mitochondria effects, T3 also increases the cell membrane permeability to Na^+^ and Ca^+^ ions and activate the Na^+^/K^+^ ATPase pump, increasing heat dissipation due to ATP hydrolysis [194].

Some studies have suggested isoform-specific actions of the TRs in regulating adaptive thermogenesis, with TRβ being involved in the induction of UCP1, while the actions of TRα potentiate beta-adrenergic signaling and induces lipolysis [195]. T3 administration reduces the activity of adenosine monophosphate-activated protein kinase (AMPK) in the ventromedial hypothalamus (VMH), leading to increased SNS output, activating BAT and UCP1-mediated thermogenesis [196]. Recently was observed that intracerebroventricular (ICV)-administered T3 also resulted in WAT browning, as expected from the mechanism of increased SNS output. Another report, that relied upon central administration of T3, came to the conclusion that “UCP1 is essential for mediation of the central effects of thyroid hormones on energy balance”, showing that ICV administration resulted in increased UCP1-mediated thermogenesis [193,197].

The beneficial effects of thyroid hormones may have on weight regulation and lipid levels are overshadowed due to poor consequences on the cardiovascular and the musculoskeletal systems. The thyromimetic compound Sobetirome has been shown to elicit a profound beigeing of WAT in genetic and diet-induced models of obesity [198]. The observed beigeing coincided with intense weight loss and anti-diabetic effects. Interestingly, all indicators of BAT activity decreased in ob/ob mice following Sobetirome administration. That is, classical BAT became deactivated while beige fat activity was increased, potentially providing a unique model by which to study the effects of WAT beigeing in the absence of effects on BAT [199]. Despite the recognized effects of thyroid hormones centrally administered with the induction of SNS activity. The systematic administration of thyroid hormones seems to exert a different action. Two recent reports have investigated the basis for thyroid thermogenesis, the increased metabolic rate observed with thyroid hormone administration, both reporting this action to be UCP1-independent [200]. T4 administered to UCP1KO mice under thermoneutral conditions, which led to a doubling of the metabolic rate. Thus, the metabolic increase observed cannot be attributed to UCP1-dependent thermogenesis in brown or beige fat. They did find, however, that chronic T4 treatment produced a substantial increase in BAT UCP1 levels, which led to a very large UCP1-dependent metabolic increase in response to NE treatment. In a similar study from Johann et al., they reported that T3 administration raised the metabolic rate, increased body temperature, resulted in weight loss, and improved glycemic control [201]. Like the aforementioned study, they also found that T3 elicited a similar response in UCP1KO mice, indicating that these effects cannot be the result of UCP1-dependent thermogenesis. While T3 administration did result in WAT beigeing, based on the induction of UCP1 and thermogenic genes, they showed further that glucose and lipid uptake is either reduced or unchanged in BAT and WAT following treatment and thus not contributing to the increased metabolism, consistent with the UCP1KO results

Estrogen may affect the adipocyte cell in different ways. In brown adipocyte cell the estrogen receptor alpha stimulation (ERα) can increase the expression of UCP1 by rising PGC-1α coactivator through AMPK. While in white adipocyte ERα activation by estrogen reduces lipoprotein lipase and increases beta-adrenergic receptor activity [202]. The glucagon-like peptide (GLP-1) may control energy metabolism through its binding to GLP-1 receptors (GLP-1R) located in both peripheral tissues (as β-pancreatic cells and vagal afferents fibers) and central nervous system. The evidence of GLP-1 central effect surge from observations of its ability to reduce food intake. However, other mechanisms of GLP-1R agonists have been shown. Liraglutide a GLP1-R agonist can activate adipose-resident invariant natural killer T (iNKT) cells, increasing the production of fibroblast growth factor 21 (FGF21), adiponectin and the activation of beigeing of white fat [203].

## 10. Potential Therapeutic Use in Humans

A characteristic of white adipocyte tissue (WAT) is the capacity to changes its dimensions. When an increase of caloric intake or reduction of physical activity produce positive energy balance the adipocyte cell become hypertrophic [54,204]. In obese and diabetic persons, the expansion through adipocyte hypertrophy is accompanied by a shift to an adverse adipokine secretory profile, which typically includes an elevated array of pro-inflammatory factors, such as TNF, IL-1β, IL-6, IL-8, resistin and monocyte chemoattractant protein 1 (MCP1), with a parallel reduction in anti-inflammatory factors, such as IL-10, adiponectin and FGF21 [205]. In obesity, WAT may become severely dysfunctional and thereby fail to appropriately expand to store surplus energy. At the whole-body level, this dysfunction results in ectopic fat deposition in other tissues that regulate the metabolic homeostasis such as hepatic, pancreatic and skeletal muscle tissues. The low inflammatory condition in the obese person reduces the insulin sensitivity and causes the majority of cardiovascular complications [17,206].

Some factors have been used as possible drugs for obesity therapy by regulating the adipogenesis of the beige adipocyte. However, many have been abandoned due to the secondary effects they induce. For example, catecholamines acting via the β3-adrenergic receptor directly stimulates the classical pathway that activates beige adipocytes. However, developing adrenergic ligands for obesity and metabolic disease applications could produce unwanted autonomic, bone, and cardiovascular effects over time. Similarly, bone morphogenetic proteins 4, 7, and 8b (BMP4, BMP7, BMP8b), atrial and brain-type natriuretic peptides, FGF21, VEGF-α, and prostaglandins, have all been shown to promote beigeing in vivo [170,172,205,207]. However, these factors may also exert potentially unwanted pleiotropic effects when translated into drugs. Indeed, the development of FGF21 mimetics was halted after phase I trials because of adverse effects [205]. New factors are being assessed as possible therapeutic targets. SLIT2 (slit homolog protein 2) that is a factor secreted from beige adipocytes, transcriptionally regulated by PRDM16, which promotes a thermogenic, PKA-dependent pathway in adipocytes and improves overall metabolic parameters in response to high-fat diet challenge [208]. The c-terminal fibrinogen like domain of angiopoietin-like 4 as a secreted factor, which induces cAMP-PKA-dependent lipolysis in white adipocytes leading the upregulation of a thermogenic program and subsequent protection against weight gain, increase in energy expenditure and improvement in glucose tolerance in high-fat diet-fed mice [209]. Recently was observed that kynurenic acid increases energy expenditure by activating G-protein-coupled receptor Gpr35, which in turn stimulates a thermogenic program in adipose tissue and increases levels of Rgs14 in adipocytes leading to enhanced β-adrenergic receptor signaling [210].

There have been many clinical studies in human adults that suggest the beneficial effect of activating beigeing from the WAT. Yoneshiro et al., [211] showed that daily 2-h cold exposure at 17 °C for 6 weeks resulted in increases in BAT activity and cold induced increments of energy expenditure and a concomitant decrease in body fat mass. Chondronikola et al. 2014 [212] reported that prolonged cold exposure for 5 to 8 h was able to increase resting energy expenditure (REE) by 15%, plasma glucose (30%) and FFA (70%) contributed to the observed increase in REE. Glucose disposal was increased in brown/beige and whole-body glucose disposal was significantly increased. In Diabetes mellitus type 2 subjects, 10 days of cold acclimation increased peripheral insulin sensitivity by 43%. Basal skeletal muscle glucose transporter type 4 (GLUT4) translocation was markedly increased and glucose uptake in skeletal muscle was increased after cold acclimation. It seems to be that cold exposure was able to increase energy expenditure and have beneficial effects on glucose metabolism supporting its role in treating obesity and related metabolic disorders in humans probably through an effect on beige activation.

Adipose-derived stem cells can be induced to differentiate to beige adipocytes in many ways, the large amount of research in this area suggests a great number of potential drugs in the next few years. Objectively, beige adipocytes in the adult have been shown to have a beneficial effect on both the sensitivity to insulin and the reduction in body weight [213]. Although the mechanisms by which these effects occur have not been sufficiently elucidated, using the modulation of the beige adipocyte in adipose cell progenitors in the adult is clearly a therapeutic approach, especially for type 2 diabetes mellitus. Taking into account that diabetes mellitus type 2 has two key pathophysiological components, the reduction of insulin secretion and the peripheral resistance to insulin action, a change in the sensitivity to insulin mediated by an increase in the number of beige adipocytes can be a great therapeutic strategy. Additionally, a reduction in body weight may lead to a reduced load on the activity of the pancreas. Studies in mouse models have demonstrated that the manipulation of the beige adipocytes is sufficient to alter energy expenditure and homeostasis.

Some experiments have shown that a reduction in glucose levels along with an increase in insulin sensitivity can be obtained with the induction of beige/brown fat in humans [212].

Certain therapeutic effects must be evaluated before the possible clinical use of beige adipocyte induction. First, it should be determined whether the metabolic effects of the beige adipocyte are subject to an increase in UCP1 or there are alternate metabolic pathways that could improve the condition of the adipose cells [76]. It is possible that the stimulated beige adipocytes, like the brown adipocytes, are not only heat generators but also contribute to the improved metabolism of glucose and lipids through the secretion of specific factors. Another necessary aspect addresses whether there is a more specific detector for the presence of beige adipocytes in the organism; although F-FDG-PET has been improved considerably, it is desirable to develop new tools or instruments that can quantify the amount of beige adipocyte tissue in the body.

The biology of beige adipocytes is so novel that it is necessary to gain an understanding of the physiological conditions, the number of days that the cells can survive, the elements that may be necessary to maintain the functionality of this cell type, and so on.

Finally, it is important to determine the specific factors that provide plasticity to beige adipose tissue, which makes possible their differentiation from a mature white adipose cell. The process of beigeing from both the precursor adipose cells as well as from the white adipose cells may be a desirable element for weight reduction.

## Figures and Tables

**Table 1:** Characteristics of different kind of adipocyte tissue.

White Adipocyte	Brown Adipocyte	Beige Adipocyte ^1^ Cold, Noradrenaline, TZD, FGF21, IL-4; IL-6
Unilocular adipocyte	Multilocular adipocytes	Unilocular → Multilocular
Lipid storage (+++)	Lipid storage (+)	Lipid storage (+++) → Lipid storage (+)
Mitochondria (+)	Mitochondria (+++)	Mitochondria (+) → Mitochondria (+++)
Fatty acid oxidation (+)	Fatty acid oxidation (+++)	Fatty acid oxidation (+) → Fatty acid oxidation (+++)
Respiratory chain (+)	Respiratory chain (+++)	Respiratory chain (+) → Respiratory chain (+++)
UCP1 (−)	UCP1 (+++)	UCP1 (−) → UCP1 (+++)
PGC-1α (+)	PGC-1α (+++)	PGC-1α (+) → PGC-1α (+++)
Markers: Resistin, ASC-1, FAB4	Markers: Zic1, Lhx8, Eva1, Pdk4, CLSTN3b	Markers: CD137, Tbx1, Cited1, Tmem26, CIDEA, CLSTN3b

^1^ Conditions that stimulate the thermogenic activity of beige adipocytes. TZD: Thiazolidinedione; FGF21: Fibroblast Growth Factor 21; UCP1: Uncoupling protein 1; PGC-1a: Peroxisome proliferator-activated receptor-gamma coactivator alpha 1; ASC-1: adipocyte–specific cell surface protein; FABP4/aP2: Fatty acid binding protein 4; Zic1: Zinc finger protein 1; Lhx8: LIM/homeobox protein; Eva1: Epithelial V-like antigen 1; Pdk4: Pyruvate dehydrogenase kinase 4; CD 137: Cluster of Differentiation 137; Tbx1: T-box transcription factor 1; Cited1: Cbp/P300-interacting transactivator 1; Tmem26: transmembrane protein 26; CIDEA: Cell death-inducing DFFA-like effector, CLSTN3b, Calsyntenin 3β. (+) : low activity; (+++): high activity.
